# European rodent on the edge: status and distribution of the Vojvodina blind mole rat

**DOI:** 10.1186/2193-1801-2-2

**Published:** 2013-01-04

**Authors:** Attila Németh, György Krnács, Virág Krizsik, Tamás Révay, Dávid Czabán, Nikola Stojnić, János Farkas, Gábor Csorba

**Affiliations:** 1 Department of Systematic Zoology and Ecology, Eötvös Loránd University, Pázmány Péter sétány 1/C, Budapest, H-1117 Hungary; 2 Kiskunság National Park Directorate, Lisz Ferenc u. 19, Kecskemét, H-6000 Hungary; 3 Hungarian Natural History Museum, Baross u. 13, Budapest, H-1088 Hungary; 4 Research Institute for Animal Breeding and Nutrition, Méhészet 1, Gödöllő, H-2100 Hungary; 5 Department in Novi Sad, Institute for Nature Conservation of Serbia, Radnicka 20a, Novi Sad, 21000 Serbia

**Keywords:** Carpathian basin, Conservation biology, Cytogenetics, Extinction, IUCN categories, *Nannospalax* (*leucodon*) *montanosyrmiensis*, Spalacinae

## Abstract

Recent research of blind mole rats of the species complex *Nannospalax* (superspecies *leucodon*) identified a small and fragmented population of these rodents on both sides of the Hungarian-Serbian border. Cytogenetic investigations proved that this population karyologically identical with the Vojvodina blind mole rat described earlier as *Nannospalax* (*leucodon*) *montanosyrmiensis*. Based on cytochrome b gene sequences obtained from three specimens originating from separate locations, these blind mole rats form a discrete phylogenetic clade which, with a difference of about 10%, is well separated from other blind mole rat taxa inhabiting the Carpathian Basin. The taxon has only two extant populations that are 150 km apart from each other. The combined occupied area is estimated to be less than 10 km^2^, and the total estimated number of individuals is less than 300. These two remaining populations are heavily fragmented and under imminent threat by the establishment of tree plantations, small-scale and agro-industrial farms and land development. The situation is further aggravated by the fact that 80% of the individuals inhabit unprotected areas. A study of the landscape history of the wider area surrounding one of the populations - based on military maps spanning over the last 200 years - has shown a drastic decrease in the extent and quality of potential habitats. Based on our present knowledge, the Vojvodina blind mole rat is one of the most seriously threatened, rarest mammal in Europe, the remaining population of which can be wiped out within years unless immediate conservation action is taken.

**Electronic supplementary material:**

The online version of this article (doi:10.1186/2193-1801-2-2) contains supplementary material, which is available to authorized users.

## Background

Rodents (Order Rodentia) are usually not in the focus of conservation biology (Lidicker 1989). Proper evaluation of the conservation status of rodent species is further complicated by the confusion that surrounds almost all levels of rodent systematics (Corti 2001). Well-established taxonomy is the base for efficient conservation biology, but unclear taxonomical questions can easily result in the negligence of certain groups, whose extinction would mean the loss of entire evolutionary lineages, drastically decreasing the overall biodiversity on Earth. The vulnerability of this order is demonstrated by the fact that rodent species contributed 51–52% to mammalian extinctions in the last 500 years (Ceballos and Brown 1995, MacPhee and Flemming 1999). Counterintuitively though, conservation initiatives will continue to be biased towards the most studied and attractive mammal groups and species (Amori and Gippoliti 2000).

The situation is clearly mirrored in the case of the Eurasian blind mole rats (Spalacidae: Spalacinae). These small mammals represent a distinct group among rodents which is extremely adapted to subterranean life. They have cylindrically shaped body with no external ear and a vestigial tail, and are completely blind spending their entire life in their tunnel system built underground (Topachevskii 1969). Compared to other rodents, the conditions resulting from their lifestyle created a decreased morphological variability and the species are very similar both externally and osteologically (Nevo 2000). Putting aside the lineage of large mole rats (genus *Spalax*) (for taxonomic context and nomenclatural details see Topachevskii 1969, Németh et al. 2009, Arslan, Akan and Zima 2011, Hadid et al. 2012), taxa belonging to *Nannospalax* present a long-standing source of dispute and disagreement on their systematics (Savić and Nevo 1990, Musser and Carleton 2005). Within the latter genus one of the recognised species groups (regarded as superspecies) which include a large number of karyologically different taxa (for the list of these named forms see Savić and Soldatović 1984) is *Nannospalax* (superspecies *leucodon*) (Musser and Carleton 2005). Molecular genetic investigations of this superspecies are quite limited so far both in terms of geographic and taxonomic coverage (Krystufek et al. 2011, Kandemir et al. 2012, Hadid et al. 2012) and the species status of taxa differentiated solely on chromosomal grounds have not been widely accepted (Sözen et al. 2006, Ivanitskaya et al. 2008). In accordance with the presently accepted view these taxa are herewith called “chromosomal forms”. Alongside with taxonomic uncertainty the determination of conservation status of different mole rat taxa is further hampered by their exclusively subterranean lifestyle which makes it difficult to evaluate their population size. While the *leucodon*-superspecies itself is categorised as Least Concern (Temple and Terry 2007), populations and habitats of many different European chromosomal forms are disappearing at an alarming rate, a phenomenon which has just recently been realized (Kryštufek and Amori, 2008; Németh et al., 2009).

In the course of researching mole rats of the Carpathian Basin, on both sides of the Hungarian-Serbian border a small and fragmented population of mole rats was identified in 2008. Cytogenetic investigations have proven that this Kelebia-Subotićka peščara population belongs to one of the four endemic chromosomal forms of the Carpathian Basin (Németh et al. 2009), *Nannospalax* (*leucodon*) *montanosyrmiensis*, described by Savić and Soldatović (1974) as *Spalax montanosyrmiensis*. This form was previously known only from two neighbouring localities at the foothills of Fruška Gora in Serbia (called as Stražilovo-Čortanovci population). In this paper we provide data for population size, distribution area and threatening factors of this poorly known chromosomal form.

## Results and discussion

### Genetic identification

Due to the methodological difficulties to culture very limited amount of blood cells for cytology we were able to karyotype a single individual only. The karyotype of a mole rat found in the vicinity of Kelebia was 2n = 54 NF = 86 consisting of 2 pairs of metacentric autosomes, 8 pairs of submetacentric autosomes, 5 pairs of subtelocentric autosomes and 11 pairs of acrocentric autosomes; the X chromosome is large and metacentric, the Y chromosome is medium sized and acrocentric. This karyotype is identical with that of previously reported from the vicinities of Stražilovo and Čortanovci in Serbia and described as *montanosyrmiensis* (Savić and Soldatović 1974, Soldatović and Savić 1983).

**Figure 1:**
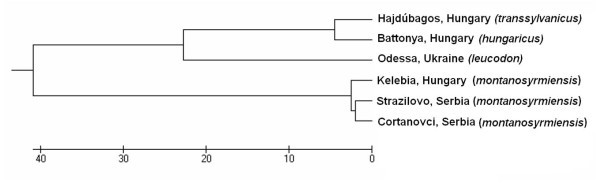
**Similarity dendrogram of Carpathian Basin blind mole rats and the nominotypical race from the type locality based on cyt b (866 bp) sequences.** The dendrogram was constructed using UPGMA method based on similarity matrix calculated with “No. of differences” (the number of different base-pairs between two compared sequence).

As we were looking for evidence of the genetic differentiation of the *montanosyrmiensis* form and not the phylogeny of the superspecies only included endemic forms from the Carpathian Basin and the nominotypical form of the superspecies (Table 1); we also refrain from taxonomic conclusions. Based on the analysed 866 bp-long part of the cytochrome b gene sequences of three individuals of Vojvodina blind mole rat the degree of between-population sequence divergence with Kimura 2-parameter distance was between 0.5 and 0.7%. This value is well below the average sister-species difference (which is more than 2.7%) in Rodentia (Bradley and Baker 2001). Thus the sequence analyses confirm the cytogenetic identity of the two populations. The samples of the two populations of the Vojvodina blind mole rat form a discrete clade (Figure 1). This clade, with the approximate 10% p-distance, is well separated from other blind mole rat taxa inhabiting the Carpathian Basin and is also clearly distinct from specimens sampled at the type locality of the lesser blind mole rat *Nannospalax* (*leucodon*) *leucodon*. Sequences generated in the present study are deposited in GenBank under Accession Nos. JN656385-JN656390; tissue samples are stored in the Mammal Collection of the Hungarian Natural History Museum (HNHM) (Table 1).

**Table 1:** **Blind mole rat specimens used in the genetic analyses**

Chromosomal form	Settlement	Country	Voucher accession no.	GenBank no.
*Montanosyrmiensis*	Kelebia	Hungary	HNHM 22789	JN656386
*Montanosyrmiensis*	Stražilovo	Serbia	HNHM 23396	JN656391
*Montanosyrmiensis*	Čortanovci	Serbia	HNHM 23397	JN656392
*Leucodon*	Odessa	Ukraine	HNHM 23208	JN656388
*Hungaricus*	Battonya	Hungary	HNHM 23001	JN656387
*Transsylvanicus*	Hajdúbagos	Hungary	HNHM 21838	JN656385

### Recent distribution

**Figure 2:**
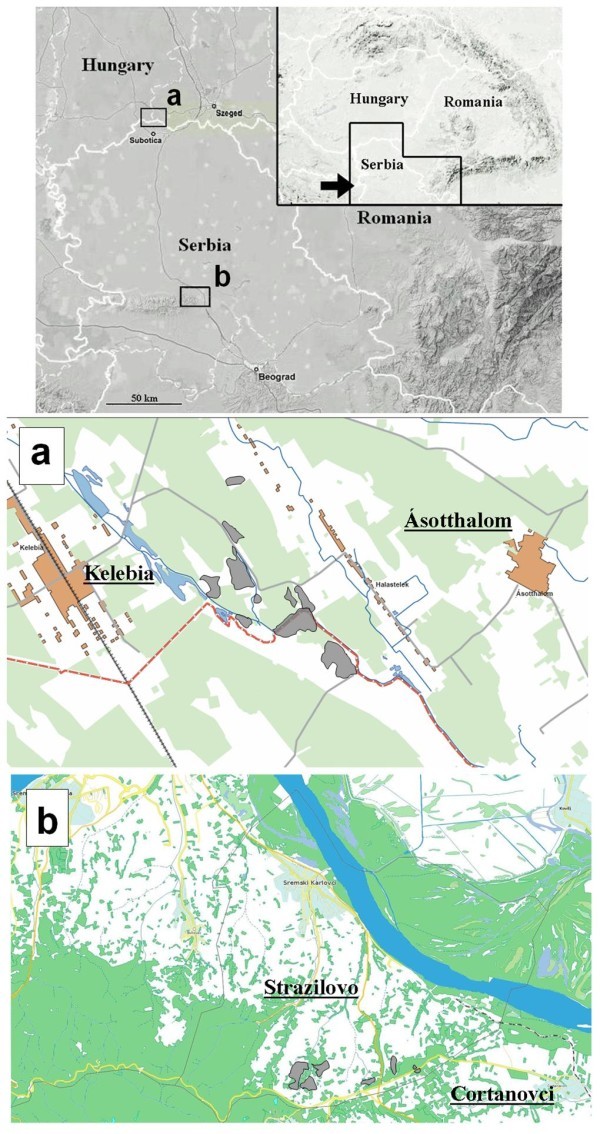
**Present distribution (shaded areas) of Vojvodina blind mole rat in the regions of a) Kelebia-Subotićka peščara and b) Stražilovo-Čortanovci.**

The Kelebia-Subotićka peščara population (Figure 2a) lives on both sides of the boundary between Serbia and Hungary. The Hungarian part (enclosed by the municipal boundaries of Kelebia) consists of 6 sub-populations isolated by roads, cultivated areas and wooded strips. These habitat fragments are small and deteriorated in quality (more than 50% of fallow land) covering a total of 16 hectares. The estimated population size in 2010 was approximately 90 individuals all together in the 6 localities. The population on the Serbian side lives within the Subotićka peščara protected area. Although this contains 400 ha of temperate grassland (sand steppe), blind mole rats only occur on a 20-hectare stretch close to the international border. Despite the fact that this habitat (regenerated steppic grassland) is of relatively good quality the population numbers only about 60 individuals (Table 2). The reason could be that almost the entire area has been ploughed in 2003 which might have affected the population seriously.

**Table 2:** **Recent populations of the Vojvodina blind mole rat**

Population	Sub-population	Extent (ha)	Area of occupancy (ha)	No. of individuals	
				2009	2010
**Kelebia- Subotićka population**	Bácsborista	5,5	2,1	12	19
	Tanyahelyek	6,9	3,1	48	15
	Smuk-ér (Tökleveles)	9,3	6,3	55	29
	Kelebiai halastavak mellett	1,1	0,5	9	10
	Kőrös-ér	2,2	4,1	24	10
	Határszeglet	2	1,5	12	7
	Subotićka peščara	400	20	50	60
	summa	427	37,6	210	150
**Stražilovo - Čortanovci population**	Stražilovo - fallow	25,2	18,3	20–23	10
	Stražilovo -meadow	13,3	10,2	15–17	16–18
	Stražilovo weekend houses	4,2	2,1	12–14	8–11
	vicinity of Čortanovci	5,7	2,9	10–12	5–7
	close to the Fruska gora- Čortanovci road	2,9	1,6	8	6-8
	Čortanovci	1,2	1	5–6	5-6
	summa	52,5	36,1	70–80	50-60
	**total population size**			**280-290**	**200-210**

The Stražilovo-Čortanovci population (Figure 2b) is situated on the slopes of Fruška gora, almost 150 km south from the previous location. Clusters of mounds were found on pastures and meadows near Stražilovo and the population extends into the village of Čortanovci where one individual was caught in a kitchen garden. Blind mole-rats occur in a landscape mosaic of small woodlands, vineyards, orchards, pastures and meadows. Many of these cultivated habitat types have turned into spontaneously regenerating fallow land with elements of loess steppic vegetation. The actual habitat of the population is about 50 ha and the estimated population size is less than 100 individuals (Table 2). No part of the habitats of this population is protected.

### Landscape history

The study area (10 × 10 km on the border of the settlements of Kelebia, Ásotthalom and Subotićka) encloses all presently known fragments of the Kelebia-Subotićka peščara population. Following the categorisation of the military surveys types of open and closed dry grasslands, dry pastures and dry hayfields are regarded herewith as potential mole rat habitats (see Topachevskii 1969, Savić and Nevo 1990, Horváth et al. 2007).

**Figure 3:**
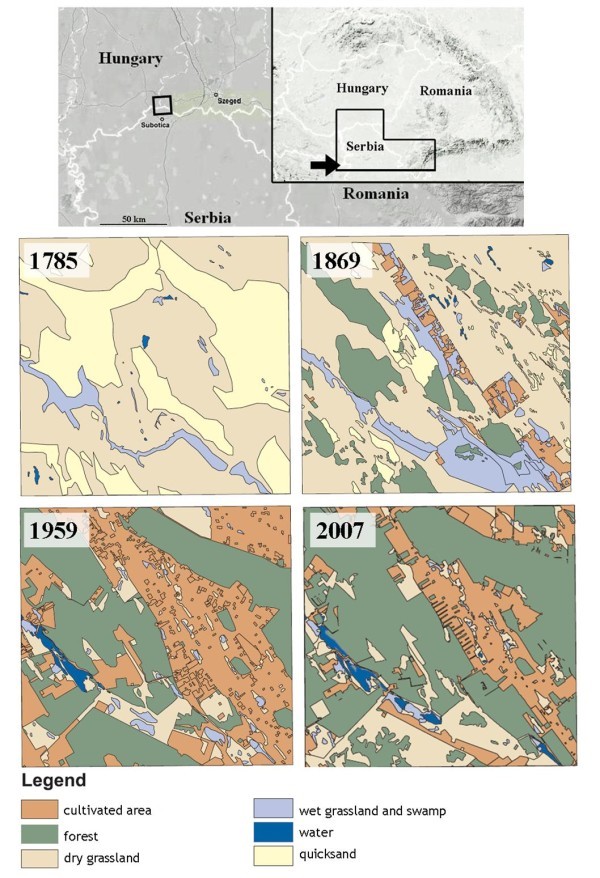
**Landscape history of the distribution area of the Kelebia-Subotićka peščara population based on four map sources (see details in text).** Note the disappearence of prime blind mole rat habitats (dry grasslands and pastures) and the steady increase of unsuitable habitat types e.g. forests and cultivated fields.

Maps from the 1st military survey (Anon. 1785) shows a treeless, uninhabited region used for grazing livestock. In that era various open or more closed grassland communities covered 61% of the area. The 2nd military survey (Anon. 1869) already shows profound changes. Spontaneously forming woodlands cover 15%, and surrounding the 67 homesteads cultivated land represents 5% of surface cover; the potential blind mole-rat habitats decreased to 45%. A next survey in 1959 (Anon. 1959) documented further increase in both woodland and cultivated area; drier grasslands formerly used as pastures were transformed by afforestation. Wooded areas totalled 35% in 1959 and their somewhat decelerating expansion is still ongoing into present days (Anon. 2007). This affected much of the remaining grasslands the extent of which shrinked to 16% only and became heavily fragmented (Figure 3).

### Risk assessment

In the Hungarian part of the Kelebia-Subotićka peščara population the major threat presently is the change in land management: tree plantations, small-scale farming and agro-industrial farming. Following the afforestation of the area in 2007, the extent of the habitat of one of the sub-populations was reduced by 50%. A small fragment (numbering less than 20%) of the population falls into a Natura 2000 site (*“Déli Homokhátság” SCI* [*Southern Sand Ridge SCI*], code: *HUKN20008*), but the rest of the habitats are not protected by any means. In the Serbian part of the Kelebia-Subotićka peščara population the major threats are small-scale wood plantations. The whole area is protected as part of the *Subotićka peščara Protected Area,* however, despite this status serious habitat destruction took place in 2003.

In the Stražilovo-Čortanovci population the major threat is rapid habitat loss. Remaining grasslands and fallow lands are directly threatened. The field where the authors caught a specimen in 2009 was ploughed in 2010 wiping out all individuals on it. The subpopulations are isolated from each other and habitat fragments are under constant threat of transformation into cultivated land. Another obstacle to effective protection is that habitats are owned by a great number of proprietors, rendering long-term protection of the total population almost impossible. Presently, none of these areas are under any kind of protection.

The *montanosyrmiensis* form is therefore proposed to be ranked as Critically Endangered according to the IUCN (2001) criteria. This assessment is based on the extent of occurrence which is estimated to be less than 100 km^2^; area of occupancy is estimated to be less than 10 km^2^. The populations are severely fragmented in no more than two locations and continuing decline is observed in area, extent and quality of habitats.

## Conclusions

This paper presents a case study where a mammal within Europe can drift to the brink of extinction almost unnoticed as a result of the lack of information, unclear taxonomic status and unrecognised tasks in conservation biology.

Karyotype of the blind mole-rat populations flanking the boundary of Serbia and Hungary were found identical to that of the *montanosyrmiensis* chromosomal form previously known only from Serbia (Soldatović and Savić 1983). The mtDNA analysis confirmed that the two populations form a discrete clade which is highly separated from other blind mole-rat taxa found in the Carpathian Basin. According to the results of the most comprehensive molecular biological research on blind mole rats based on 6 mitochondrial genes, the *montanosyrmiensis* form is a well separated lineage that diverged from the closest taxon examined about 1.8 million years ago (Hadid et al. 2012). Therefore, *montanosyrmiensis* complies with the criteria of the *Genetic Species Concept* (Baker and Bradley 2006), as well as the *ESU* (*Evolutionary Significant Unit*) (Moritz 1994).

Based on the surveys of all potential habitat patches in the presumed distribution area, the taxon *montanosyrmiensis* has only two extant populations that are 150 km apart from each other and the total estimated number of individuals is less than 300. Even these two remaining populations are heavily fragmented and extensive unsuitable vegetation types (forests, arable lands) between suitable habitat patches - often smaller than 3 hectares – possibly obstruct recent gene flow between subpopulations. The status and future of these population fragments are precarious as 80% of the individuals inhabits areas with no protection of any form, while constantly being threatened by factors such as ploughing, afforestation and site development. As the landscape history study revealed the Kelebia-Subotićka peščara population has lost more than 70% of its potential habitat in the last 200 years.

Based on our present knowledge, the Vojvodina blind mole rat is one of the most seriously threatened, rarest mammal in Europe, the remaining population of which can be wiped out within years unless immediate conservation action is taken.

## Methods

### Distribution and population size

**Figure 4:**
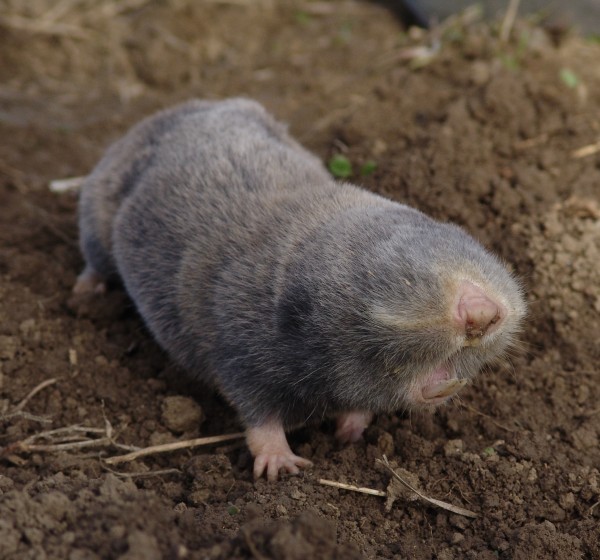
**Vojvodina blind mole rat (*Nannospalax*(*leucodon*)*montanosyrmiensis*) from Čortanovci, Serbia.**

**Figure 5:**
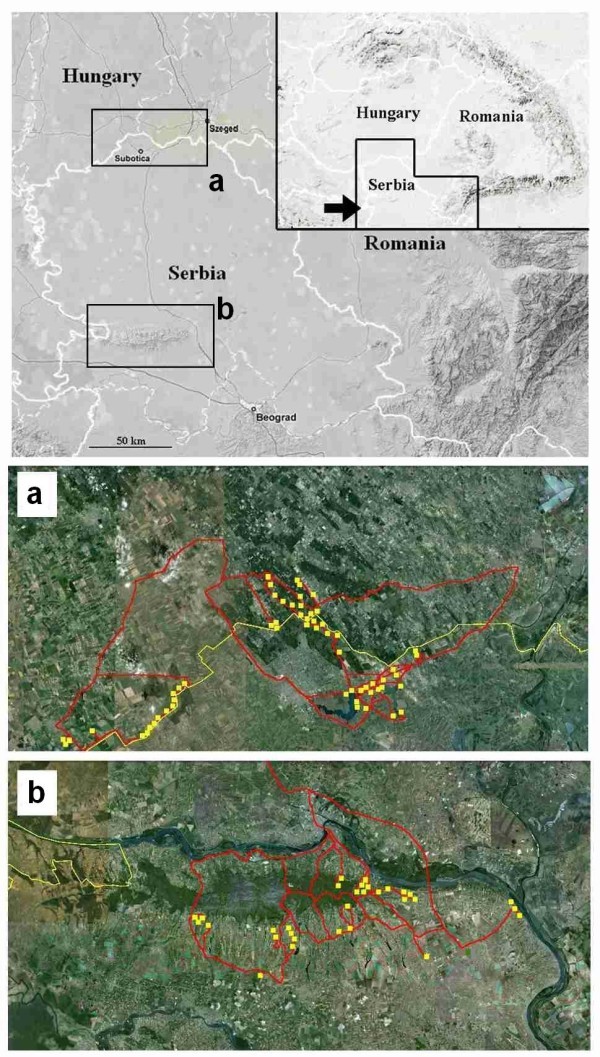
**Maps of investigated areas around the known populations (a and b) of Vojvodina blind mole rat.** On the detailed maps yellow lines show international borders; red lines indicate the accessed routes; whereas yellow squares mark the throughoutly investigated potential habitat patches.

Between 2008 and 2010 we systematically evaluated all known Hungarian and Serbian localities of Vojvodina blind mole rats (Figure 4) and also covered the remaining grasslands and fallow lands within its supposed distribution area (Figure 5, Additional file 1). Only locations that were confirmed by the authors are listed below as “Recent distribution”. Population size estimations were based on consistently monitoring the location of fresh mounds (Zuri and Terkel 1997). Since in all localities the clusters of mounds (one cluster is the visible sign of the underground activity of a single animal) were clearly separated from each other in this way it was possible to distinguish between mounds that belonged to different individuals. Counts were carried out in spring and in autumn months (during the peak activity periods of blind mole rats) in every locality at least in two consecutive years (Additional file 1). A population was defined as a set of sub-populations (inhabiting a more or less continuous, and homogeneous habitat) which were not separated from each other by impassable barriers (marshes or rivers). Gene flow between the two populations (i.e. the Kelebia-Subotićka peščara population and the Stražilovo-Čortanovci population) is quite improbable due to their distance and separating unsuitable habitats.

Coordinates of all clusters of mounds were recorded by a hand-held GPS device. GPS records outlined the total extent of the distribution area. Spatial information, such as size and distribution of habitat patches, annual changes in the geographical position of mounds was tracked by means of Google Earth 5.1 and ArcGIS 9.0.

### Sampling

As the Vojvodina blind mole rat is strictly protected both in Hungary and Serbia only 1-3 specimens were caught alive per sub-populations to prove the presence of the animals as allowed by research permits issued by the National Inspectorate for Environment, Nature Conservation and Water no. 14/05173-3/2006 and 14/1840-3/2008. Altogether 16 animals were captured and handled in the field in accordance with guidelines approved by the American Society of Mammalogists (Gannon et al. 2007). The animals were caught by opening the tunnel system and capturing the animal trying to mend the damage (Németh et al. 2007). After samples were taken for tissue cultivation and DNA-analysis, individuals were released at the site of capture straight into their own tunnels. For cytogenetic investigations, instead of using the lethal direct bone marrow preparation (widely used in rodents) we took blood samples aided by veterinarians, applying topical and systemic aenesthesia and 70% alcohol disinfection following the protocoll described by Sós et al. (2009). Lymphocyte cultures were used for chromosomal investigations. Metaphase arrest, hypotonic treatment and chromosome preparation were carried out according to standard cytogenetic techniques (Moorhead et al.^1960^) with using RPMI 1640 medium containing 20% FCS, antimycotic, antibiotic solutions and the mixture of phytohaemagglutinin and pokeweed mitogen (1-1%).

### Sequence analysis

The main objective of the molecular biological investigation was to set the Kelebia-Subotićka peščara population and the Stražilovo-Čortanovci population in genetic comparison. Genomic DNA was isolated from fresh tissue samples with Qiagen DNeasy Blood and Tissue kit (Qiagen, California, USA) according to the extraction protocol. The 866 bp long sequences of cytochrome *b* was amplified with PCR using forward F-muarso (5′-ATGACATGAAAAATCATYGTTGT-3′) and reverse R-muarso (5′-GAAATATCATTCKGGTTTAATRTG-3′) primer pairs. The polymerase chain reaction was performed in total volume of 25 μL containing 30 ng template DNA, 1 μM of each oligonucleotide primer, 1.5 mM MgCl_2_, 160 μM dNTPs and 0.5U AmpliTaq DNA polymerase (Applied Biosystems). PCR amplification was conducted in a DNA Engine Dyad (MJ Research) employing 94°C for 2 min followed by 45 cycles of 94°C for 45 sec, 48°C for 15 sec (ramp speed down to 48°C: 1C/sec), 60°C for 1 sec (ramp speed: 0.5C/sec up to 60°C) and 72°C for 2 min (ramp speed: 1C/sec up to 72°C), and the final extension step was 72°C for 7 min. The PCR-product was checked on 1.6% agarose gel stained with ethidium bromide and cleaned with Qiagen Clean Up kit (Qiagen, California, USA).

The A-tailing reaction was contained 7 μL of PCR product, 200 μM dATP, 1.5 mM MgCl2, 1x Taq buffer and 5U AmpliTaq DNA polymerase. The tailing profile was 94°C for 3 min and 70°C for 30 min. The A-tailed PCR product was ligated into pGEM Easy vector (Promega) and transformed into JM109 cells. Positive colonies were applied as a template for reamplification by PCR utilizing the T7 and SP6 primers. The products were checked on 1.6% agarose and then cleaned with SureClean (Bioline). The sequencing reaction was accomplished in GeneAmp PCR System 9700 machine according to the thermal profile of 94°C for 4 min followed by 25 cycles of 94°C for 30 sec, 50°C for 15 sec and 60°C for 4 min. The 10 μL sequencing reaction consisted of 80 ng template, 2 μL of BigDye v3.1 Terminator, 1 × buffer and 1 μM primer and then products were cleaned using the BigDye XTerminator Purification Kit. Sequences for both directions were obtained using ABI3130 Genetic Analyser and were aligned and tested in MEGA v5 (Tamura et al. 2011). Prior to the analysis the aligned sequences were checked for the presence of stop codons by using ORF finder at NCBI (http://www.ncbi.nlm.nih.gov/gorf/gorf.html). Phylogenetic relation of the samples was reconstructed using UPGMA method based on similarity matrix calculated with “no. of differences” (the number of different base-pairs between two compared sequence) and “kimura 2-parameter” technique. P-distance was obtained by dividing the number of nucleotide differences by the total number of nucleotides compared.

### Landscape history

The landscape history could only be investigated on the two remaining populations of the Vojvodina blind mole rat but historical maps covering the last two centuries are available for the Kelebia-Subotićka peščara population only. These historical sources, which contain information on the changes of the landscape and of land use both in space and time, were the relevant digitized chart pages of military maps from the 1st (Anon. 1785) and 2nd (Anon. 1869) Military Mapping Survey of Austria-Hungary, and from the map series published by Honvéd Mapping Institute (Anon. 1959). Changes in land use during the past 50 years were assessed based on aerial photography (Anon. 2007). To track and interpret changes in landscape history ArcGIS 9.0 software was used.

### IUCN classifications and categories

The Red List categories were assessed according to the 2001 criteria (IUCN 2001). Habitats and threats were classified (and terms used) according to the IUCN Habitats Classification Scheme 3.0 and Threats Classification Scheme 2.1, respectively (http://www.iucnredlist.org).

## Electronic supplementary material

Additional file 1: **Surveyed potential sites within the presumed distribution area of the Vojvodina blind mole rat.** (PDF 23 KB)
