# Supplementary material for: European rodent on the edge: status and distribution of the Vojvodina blind mole rat
Source: Springerplus. 2013 Jan 4;2:2. doi: 10.1186/2193-1801-2-2 (PMC3579411; doi:10.1186/2193-1801-2-2)
Supplement: Supplementary file 1 — Additional file 1: Surveyed potential sites within the presumed distribution area of the Vojvodina blind mole rat. (PDF 23 KB) [file 40064_2012_76_MOESM1_ESM.pdf]

Online Resource 1.

Surveyed potential sites within the presumed distribution area of the Vojvodina blind mole rat

Site(s)	Closest settlement	Country	Centered at		Extent (ha)	Date of survey
			Longitude (N)	Lattitude (E)		
Bácsborista	Kelebia	Hungary	46°13,309'	19°40,284'	5,5	October of 2008
						October of 2009
						October of 2010
Tanyahelyek	Kelebia	Hungary	46°12,642'	19°40,200'	6,9	March of 2008
						October of 2009
						October of 2010
Smuk-ér (Tökleveles)	Kelebia	Hungary	46°11,823'	19°40,548'	9,3	March of 2008
						October of 2009
						October of 2010
Kelebiai halastavak	Kelebia	Hungary	46°11,489'	19°39,742'	1,1	March of 2008
						October of 2009
						October of 2010
Kőrös-ér	Kelebia	Hungary	46°11,332'	19°41,858'	2,2	March of 2008
						October of 2009
						October of 2010
Határszeglet	Kelebia	Hungary	46°11,155'	19°40,560'	2	March of 2008
						October of 2009
						October of 2010
Emlékerdő	Ásotthalom	Hungary	46°12,905'	19°47,488'	15	May of 2008
Öttömösi baromjárás	Öttömös	Hungary	46°15,369'	19°41,567'	104	May of 2008
Sztipityjárás	Kelebia	Hungary	46°13,845'	19°37,108'	76	October of 2008
Szkenderjárás	Kelebia	Hungary	46°11,550'	19°38,906'	60	May of 2008
						October of 2008
border zone	Madaras, Kunbaja, Csikéria	Hungary -Serbia	46°02,127'	19°22,858'	7,6	March of 2009
Kolbászsor	Vasútföld	Hungary	46°15,520'	19°33,433'	250	October of 2008
grassland in the vicinity of…	Újföld	Hungary	46°13,765'	19°35,357'	3,5	October of 2008
grassland in the vicinity of…	Kelebia	Hungary	46°12,499'	19°37,693'	39	May of 2008
Iskola-dűlő	Öttömös	Hungary	46°17,047'	19°39,904'	43	October of 2008
grassland in the vicinity of…	Madaras	Hungary	46°02,297'	19°16,737'	29	March of 2009
Suboticka pescara	Suboticka (Szabadka)	Serbia	46°10,620'	19°42,519'	400	May of 2008
						October of 2009
						October of 2010
grassland in the vicinity of…	Donji Tavankut (Alsótavankút)	Serbia	46°04,900'	19°28,117'	150	March of 2009
Szelevényi puszták	Szelevény	Serbia	46°08,728'	19°51,989'	667	May of 2008
mosaics of grasslands, fallow lands and gardens in the vicinity of…	Hajdúkovo (Hajdújárás)	Serbia	46°06,992'	19°49,758'	1015	May of 2008
						October of 2008
						October of 2009
	Királyhalom	Serbia	46°07,702'	19°52,136'	2026	October of 2008
	Palić (Palicsfürdő)	Serbia	46°05,529'	19°46,647'	2002	October of 2008

						October of 2009
	Ludus (Ludas)	Serbia	46°05,653'	19°50,224'	850	October of 2008
						October of 2009
	Strazilovo	Serbia	45°09,410'	19°56,237'	129	October of 2009
	Cortanovci	Serbia	45°09,463'	19°57,766'	28	October of 2009
	Bukovac (Bakolc)	Serbia	45°10,875'	19°52,994'	93,6	October of 2009
						October of 2010
	Beška	Serbia	45°07,440'	19°02,581'	38,5	October of 2009
						October of 2010
	Jazak	Serbia	45°05,525'	19°45,729'	10,7	October of 2009
						October of 2010
			45°05,221'	19°45,752'	7,2	October of 2009
						October of 2010
			45°05,120'	19°45,990'	6,4	October of 2009
						October of 2010
	Mala Remeta	Serbia	45°05,443'	19°44,575'	25,5	October of 2009
						October of 2010
	Šatrinci (Satrinca)	Serbia	45°03, 910'	19°55,368'	3	October of 2009
						October of 2010
	Grgeteg	Serbia	45°07,919'	19°54,116'	44	October of 2009
						October of 2010
	Neradin	Serbia	45°06,547'	19°54,109'	13,5	October of 2009
						October of 2010
	Krčedin (Kereked)	Serbia	45°08,603'	20°08,484'	3	October of 2009
						October of 2010
	Krušedol	Serbia	45°07,690'	19°56,735'	18	October of 2009
						October of 2010
	Stari Slankamen (Szalánkamén)	Serbia	45°09,657'	20°13,842'	5	October of 2009
						October of 2010
			45°08,233'	20°15,218'	0,8	October of 2009
						October of 2010
			45°08,261'	20°15,442'	1,3	October of 2009
						October of 2010
	Golubinci	Serbia	45°58,702'	20°03,299'	8	October of 2009
						October of 2010
	Stejanovci	Serbia	45°02,672'	19°43,184'	37	October of 2009
						October of 2010
	Lezimir	Serbia	45°07,695'	19°35,295'	50	October of 2009
						October of 2010
			45°06,637'	19°34,261'	19,6	October of 2009
						October of 2010
